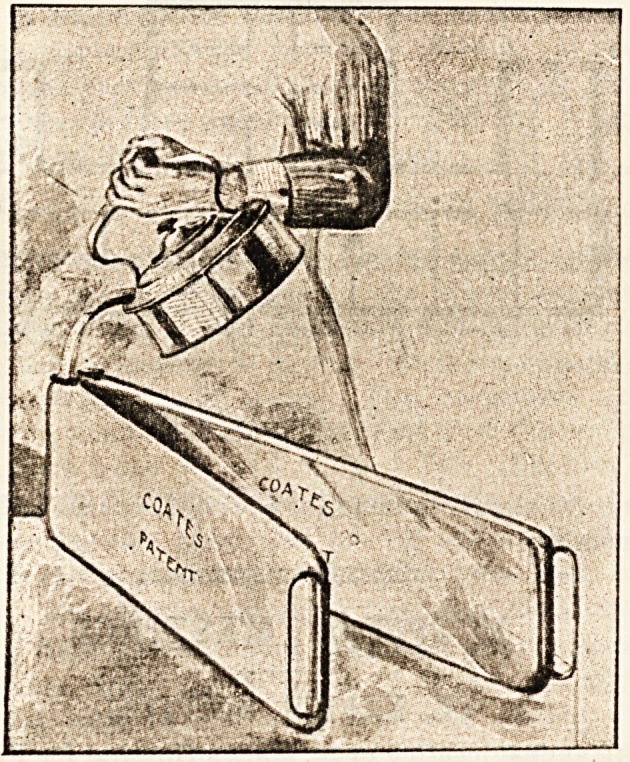# Practical Departments

**Published:** 1905-02-18

**Authors:** 


### PRACTICAL DEPARTMENTS.

### COATES' PATENT BED AND FOOT-WARMER.

(14 Thavies' Inn, E.C.)

This new type of bed-warmer may be recommended to the notice of those in charge of hospital wards or of private nursing institutions. As will be seen from the illustration, the apparatus consists of two halves jointed together by a hinge. When opened out the whole warmer measures four feet; it thus serves admirably for the purpose of warming the bed previous to the reception of a patient, especially after a prolonged operation. The ordinary hot-water bottles are apt to warm only a small part of the bed, and the necessity for warmth in the case of patients who are in a state of collapse needs no emphasising in these days. By removing the pin of the hinge the two sections may be used separately and each half, well covered with a flannel jacket, makes an excellent "hot bottle." For carriage or motor-car no better foot-warmer could be devised, and the price is moderate. When not in use the warmer takes up very little room, each section being only ¾-inch thick.

## Figures and Tables

**Figure f1:**